# Thyroid-Gut-Axis: How Does the Microbiota Influence Thyroid Function?

**DOI:** 10.3390/nu12061769

**Published:** 2020-06-12

**Authors:** Jovana Knezevic, Christina Starchl, Adelina Tmava Berisha, Karin Amrein

**Affiliations:** 1 Division of Endocrinology and Diabetology, Department of Internal Medicine, Medical University of Graz, Auenbruggerplatz 15, 8036 Graz, Austria; jovana.knezevic@stud.medunigraz.at (J.K.); karin.amrein@medunigraz.at (K.A.); 2 Department of Psychiatry and Psychotherapeutic Medicine, Medical University of Graz, 8036 Graz, Austria; adelina.tmava@medunigraz.at

**Keywords:** thyroid, gut microbiota, Hashimoto’s thyroiditis, Grave´s disease, celiac disease, Non-celiac wheat sensitivity, iodine, iron, probiotics, thyroid cancer

## Abstract

A healthy gut microbiota not only has beneficial effects on the activity of the immune system, but also on thyroid function. Thyroid and intestinal diseases prevalently coexist—Hashimoto’s thyroiditis (HT) and Graves’ disease (GD) are the most common autoimmune thyroid diseases (AITD) and often co-occur with Celiac Disease (CD) and Non-celiac wheat sensitivity (NCWS). This can be explained by the damaged intestinal barrier and the following increase of intestinal permeability, allowing antigens to pass more easily and activate the immune system or cross-react with extraintestinal tissues, respectively. Dysbiosis has not only been found in AITDs, but has also been reported in thyroid carcinoma, in which an increased number of carcinogenic and inflammatory bacterial strains were observed. Additionally, the composition of the gut microbiota has an influence on the availability of essential micronutrients for the thyroid gland. Iodine, iron, and copper are crucial for thyroid hormone synthesis, selenium and zinc are needed for converting T4 to T3, and vitamin D assists in regulating the immune response. Those micronutrients are often found to be deficient in AITDs, resulting in malfunctioning of the thyroid. Bariatric surgery can lead to an inadequate absorption of these nutrients and further implicates changes in thyroid stimulating hormone (TSH) and T3 levels. Supplementation of probiotics showed beneficial effects on thyroid hormones and thyroid function in general. A literature research was performed to examine the interplay between gut microbiota and thyroid disorders that should be considered when treating patients suffering from thyroid diseases. Multifactorial therapeutic and preventive management strategies could be established and more specifically adjusted to patients, depending on their gut bacteria composition. Future well-powered human studies are warranted to evaluate the impact of alterations in gut microbiota on thyroid function and diseases.

## 1. Introduction

Humans have always been living together in a symbiotic community with their microbiota [1]. Trillions of bacteria are found in the human intestine, in jejunum and ileum, but most of them are in the colon, where the two dominant strains are *Firmicutes* and *Bacteroides* [2]. The bacterial composition shows geographical differences and numerous factors influence the composition of the microbiota, starting in utero [3] and continuing with the way a baby is born and if it is being breastfed [4]—children born by Caesarean section have a lower diversity of intestinal bacteria [3]. This extends to the influence of diet, use of antibiotics, other drugs, genetics, environment, and diseases [1,5]. Microbiota seem to reach the maturity of an adult at the age of about three years, however this can be changed at any age by the factors mentioned above [1].

Intestinal bacteria play a role in vitamin synthesis (vitamin K, folic acid, vitamin B2, B3, B5, B6, B7, and B12 [6,7], digestion of dietary fibers, regulation of the immune response, and mental disorders [8]. In regard to nutrition, the composition of the microbiota can be positively influenced by dietary fibers and other probiotic factors. For example, a rodent study showed that a change from a low-fat and high-fiber diet to a “Western diet” (high sugar, high fat, low fiber) made a difference in their microbiota composition after just one day [5]. David et al. illustrated changes in the microbiota in 10 participants after only five days of eating either a plant-based or animal-based diet [9]. Dietary fibers are of great importance to the intestine as their fermentation and the resulting short chain fatty acids (SCFAs) serve as an energy source for the enterocytes [5,10]. In addition, SCFAs (especially butyrate) impact the immune regulation and have anti-inflammatory effects [10,11].

The negative influence on the immune system and the inflammatory regulation of an impaired microbiota seems to be likely to promote autoimmune diseases such as autoimmune thyroid diseases (AITD) [4]. Hashimoto’s thyroiditis (HT) is the most common thyroid disorder worldwide with a general prevalence of around 10–12% and is characterized by chronic inflammation, autoantibodies against thyroid peroxidase (TPO) and thyroglobulin (TG), leading to hypothyroidism, and often, destruction of the thyroid gland [12,13]. Graves’ disease (GD) has a prevalence of 1–1.5% and is marked by autoantibodies against the thyroid stimulating receptors (TSHR), causing hyperactivity of the thyroid [14,15]. The consequences of these two AITDs affect the entire metabolism of the human body. Graves orbitopathy is the most relevant extrathyroid manifestation [10,16].

Although the exact mechanisms behind AITDs have not been clarified yet, it has been proposed that the interaction of genetic predisposition, immune impairment, and environmental factors (i.e. micronutrients, gut microbiota) play an important role in the pathogenesis of these diseases. HT often requires a lifelong hormone replacement therapy with levothyroxine and GD patients usually need thyreostatic drugs like propylthiouracil and methimazole and often also radioiodine therapy or surgery to control the disease in the long term [12,15]. There is increasing evidence for the presence of an important thyroid–gut axis that modulates these autoimmune diseases and patients often report changes in their quality of life and thyroid function in relation to dietary changes.

## 2. Intestinal Influences on the Thyroid

The gut microbiota largely regulates the homeostasis as well as the development of immune cells. It modulates both the innate and the adaptive immune system, even outside the gut [5], and is fundamental in the development of gut-associated lymphatic tissue (GALT), where more than 70% of the entire immune system is situated [1]. GALT plays an important role in the development of tolerance to self-antigens by controlling its toll-like receptors (TLR) in the intestinal mucosa [2].

There is a positive correlation between the short chain fatty acid butyrate concentration and the number of regulatory T-cells (TREGs), which are key mediators of immune tolerance, just as with lower concentrations of the proinflammatory Th-17 cells [10,17]. SCFAs are able to strengthen intercellular tight junctions together with thyroid hormones [4]. The immune system itself has an influence on the composition of the gastrointestinal microbiota, which underlines the symbiotic relationship. In germ-free (gf) mice, disturbed maturation of immune cells was found due to a lack of microbial stimulus to the immune system. Furthermore, shortened villi and crypts, changes in permeability and a thinner mucous membrane layer were reported in these animals [1,2,18].

The signs of immunodeficiencies in gf mice include a reduced number of T-helper cells (specifically CD4 + Th cells), reduction of Th-17 and TREG differentiation, and reduced production, respectively [1,11]. All of these immune cells play a role in the pathogenesis of AITD like HT and GD, as well as in the intestinal autoimmune Celiac Disease (CD) and Non-celiac wheat sensitivity (NCWS) [19,20]. Even though NCWS usually does not show autoimmunity, it reveals several similarities in the pathogenesis with the autoimmune diseases mentioned above [21]. An alteration in the composition of intestinal bacteria (dysbiosis), a bacterial overgrowth increasing intestinal permeability, and a shift to proinflammatory cells are some of the factors of microbial impact on the thyroid [4,19]. Zhao et al. and Ishaq et al. investigated the microbial composition in euthyroid and hypothyroid HT patients and found a dysbiosis as well as a bacterial overgrowth in the hypothyroid patient group [22,23]. Lauritano et al. assigned the bacterial overgrowth in connection with hypothyroidism mainly to the small intestine [24].

Iodothyronine-deiodinases play an important role in the conversion of thyroxine (T4) to its active form triiodothyronine (T3) or reverse T3 (rT3), its inactive form [4]. Deiodinase activity has also been found in the intestinal wall and could contribute to total T3 body levels. A study conducted in rats showed binding of thyroid hormones by gut bacteria and even competing with albumin [1,25].

Another influencing factor of microbiota is its effect on neurotransmitters such as dopamine, which can inhibit thyroid stimulating hormone (TSH). A 25% higher TSH secretion was demonstrated in gf mice [26].

### 2.1. Celiac Disease

Celiac disease (CD) is an intestinal autoimmune disease where the gluten’s gliadin fraction triggers a relevant immune response [27]. Gliadin, potentiated by tissue transglutaminase (TG2), binds to HLA-DQ2/DQ8 antigen-presenting cells and hence induces a CD4 + T cell-mediated cascade [28]. While 95% of celiac disease patients are HLA-DQ2 positive, only about 5% are HLA-DQ8 positive [29].

On the one hand, the activated CD4 + T cells cause a cytokine-mediated inflammatory reaction, lure cytotoxic CD8 + T cells [30], which destroy inflamed enterocytes and on the other hand, they activate B-cells. The B cells then start producing antibodies against gliadin, the body’s own tissue transglutaminase (anti-TG2-Ab), and endomysium (anti-EMA) [28]. All of this results in severe inflammation of the intestinal mucosa, atrophy of the villi in the small intestine, and increased intestinal permeability [27]. Symptoms can vary and range from malabsorption and gastrointestinal discomfort (diarrhea, constipation, bloating) to extraintestinal manifestations such as iron deficiency, weight loss, chronic fatigue, and other non-specific symptoms, which may also resemble and overlap those of hypothyroidism [28].

### 2.2. Non-Celiac Wheat Sensitivity (NCWS)

NCWS is defined as a non-allergic, non-autoimmune disease in which gluten consumption can lead to symptoms similar to those of celiac disease without specific immunologic findings (21). However, NCWS also has immune-related origins, indicating activation of the innate immune system. This can be shown by an increased expression and activation of toll-like receptors (TLRs), such as TLR 2 and TLR 4, and TNF-α [21,29]. The symptoms, which typically occur shortly after the consumption of gluten-containing foods, include intestinal disorders such as those found in CD, fatigue, anemia, and brain fog. It is more and more taken into consideration that gluten does not seem to be the only cause of discomfort in wheat-sensitive patients [29]. Other grain proteins, such as amylase trypsin inhibitors (ATI), may also act as triggers [31]. For this reason, instead of “non-celiac gluten sensitivity”, the more appropriate term appears to be “non-celiac wheat sensitivity” [32].

Multiple studies showed a higher prevalence of coexisting AITDs in CD [33,34], as well as in NCWS [35,36] patients and vice versa. Different hypotheses connect these diseases by discussing (I) shared cytokines in pathogenesis pathways [37,38,39], (II) cross-reaction of antibodies (molecular mimicry) [19,40], (III) malabsorption of essential micronutrients for the thyroid [41] (e.g., iron, vitamin D, and selenium [42,43,44] in CD, and (IV) increased intestinal permeability (IP) as a result of damaged intestinal intercellular junctions [45,46].

In further consequence, harmed tight junctions (TJ) lead to penetration and exposure of pathogens to the immunoreactive sub-epithelium, promoting inflammation and autoimmunogenesis [45,46]. Zonulin, a protein that when triggered by gluten or certain bacteria, can be secreted by the small intestine and modulate the integrity and permeability of intercellular junctions. When gluten induces zonulin release, it weakens the connection between the tight junctions and breaks the intestinal barrier. In general, zonulin expression is increased in autoimmune diseases [47]. Paterson et al. compared a zonulin peptide inhibitor AT1001 with placebo in a randomized clinical trial including 21 patients with CD. The results revealed that the permeability increased by 70% in the placebo group and the proinflammatory cytokine release decreased by 28% in the AT1001 group [48]. Some of the cytokines involved in inflammatory processes due to increased pathogen exposure to the intestinal immune cells are TNF-α und INF- γ [46,49]. Among others, these can also be involved in the pathogenesis of HT, GD, CD, and NCWS [21,37,38,39].

The local inflammation adds to the issue by increasing the intestinal permeability itself [46]. The intestinal permeability is influenced by various factors. Drugs (e.g., proton-pump inhibitors, non-steroidal anti-inflammatory drug), stress, alcohol, bacteria, cytokines, reactive oxygen species, and microbial dysbiosis have a negative effect on TJ integrity [50]. In contrast, glutamine [51], polyphenols (contained in turmeric, green tea, citrus fruits, etc.) [52], and vitamin D help to maintain TJ function [49,50,53]. Kong et al. showed increased mucosal damage in vitamin D receptor knockout mice, implicating a protective effect of vitamin D on the intestinal mucosa [54]. Probiotics also seem to support the intestinal barrier function positively [45].

### 2.3. Micronutrients

Microbiota influence the uptake of minerals relevant to the thyroid including iodine, selenium, zinc, and iron. All of them play a role in supporting thyroid function and there is a clear link between thyroid dysfunction and altered levels of these minerals.

Beyond that, there seems to be a negative correlation between *Lactobacillaceae* and *Bifidobacterium* spp. with dietary iron and a positive correlation with selenium and zinc. As these bacteria are diminished in Hashimoto thyroiditis and Grave´s disease, it has been suggested that gut composition and mineral regulation may have an impact on these diseases [4].

#### 2.3.1. Iodine

Iodine is essential for the synthesis of thyroid hormones and an average adult body contains around 15 to 20 mg of iodine, mainly located in the thyroid gland. Iodine is absorbed in the stomach, duodenum, and jejunum. Uptake is accomplished by the sodium/iodide symporter (NIS), which is not only expressed in thyroid cells, but also in extrathyroidal tissues including salivary gland, breast tissue, and in the stomach. In the gastrointestinal tract, iodine uptake is mediated by intracellular iodine concentrations. Besides NIS, in the gut, it can be additionally absorbed via sodium multivitamin transporter (SMVT) and cystic fibrosis transporter (CFTR); however, only to small extents [4,55,56]. In inflammatory bowel disease (IBD), a reduction in the diversity of gut microbiota and a lower abundance of *Firmicutes* and *Bacteroidetes* have been observed. Iodine malabsorption is a common consequence of IBD and vice versa, suggesting a reciprocal relationship. Chronic inflammation promotes changes in the composition of the microbiota due to alterations in the oxidative and metabolic environment of the intestine [4,57].

The amount of iodine in soil determines the iodine content of food, resulting in regional differences. Seafood and seaweed, especially from saltwater, are a rich iodine source. Thus, regions near to the ocean and cultures with high seafood consumption, like the Japanese, [58] are more likely to be iodine sufficient. However, iodine fortification of salt and milk products attempts to ensure better overall global access to iodine sources [59].

Thyroid hormone synthesis is affected by goitrogens, which are substances that either inhibit iodine uptake in the thyroid or synthetization of iodine compounds, including thiocyanate and perchlorate, which compete with iodine for the NIS [60]. Aside from that, iodine uptake is influenced by numerous substances including humic acids, fluorides, nitrates, ferrous sulfate, sucralfate, and aluminum hydroxide. In addition, soy, phenobarbital, phenytoin, carbamazepine, rifampicin, propranolol, amiodarone, and glucocorticoids interfere with thyroid metabolism and organification of iodide [4,61,62].

Iodine deficiency can lead to goiter, probably thyroid nodules, and even thyroid cancer. On the other hand, papillary thyroid cancer seems to be more common in areas with high iodine intake, suggesting complex relations between iodine levels and adverse outcomes [63,64]. Iodine—at least when applicated during medical procedures in high doses—on the opposite, has been proven to influence the gut microbiota. Administering iodine containing contrast agents can have noxious effects on the microbiota by binding to the amino acids tyrosine and histidine on the bacterial membrane, as well as by oxidation of cytoplasmic and membrane components [4].

Excess intake of iodine triggers the Wolff-Chaikoff effect, a transient reduction of thyroid hormone synthesis for around 24 h after ingestion of a high iodine load [65]. High iodine intake additionally can either induce hypothyroidism in susceptible patients, such as those with autoimmune thyroid disease, antithyroid drug therapy, or patients with higher intake of goitrogens, but it can also cause hyperthyroidism in patients at risk, e.g., with diffuse nodular goiter or latent Grave´s disease [66].

#### 2.3.2. Iron

Dietary non-heme iron (Fe^3+^) absorption is improved by acidic pH and mainly occurs in the proximal duodenum by divalent metal ion transporter 1 (DMT1), after being reduced by duodenal cytochrome b to Fe^2+^ [4,67]. On the contrary, heme iron Fe^2+^, an important source for both the human and the intestinal microbiota, is directly absorbed by heme/folate transporter 1 (HCP1) in the host and by siderophores like enterobactin in bacteria. Particularly pathogenic strains grow well in heme-rich conditions, due to their efficient heme capturing ability [68]. Many enteric gram-negative bacteria, including *Salmonella*, *Shigella*, and pathogenic *E. coli* require iron for their virulence and colonization [69,70]. Beneficial commensal gut bacteria from genera *Lactobacillus* and *Bifidobacterium* on the other hand require less or no iron [71]. In mice, Constante et al. demonstrated that a heme-rich diet decreases microbial diversity and increases the abundance of *Proteobacteria,* namely *Clostridiales and Lactobacilales.* A heme-rich intestinal environment may favor bacteria-coding genes linked to heme uptake [72].

On the one hand, iron is essential for bacterial growth and iron availability influences the composition of the microbiota because some bacteria have developed better heme-catching mechanisms. On the other hand, microbiota are able to increase iron bioavailability in the colon through lowering of pH via the production of short chain fatty acids. Bacteria possess siderophores, such as enterobactin, which are high-affinity proteins for iron that acquire Fe^3+^, especially in iron-poor environments. Humans developed a defense protein called lipocalin-2 to sequester siderophores and limit microbial growth [4,68,73].

Administration of iron supplements (due to incomplete absorption of around 20%) increases colonic iron, leading to adverse effects and altering the microbiota. In vitro and in vivo studies proposed that through oral iron, the beneficial barrier of commensal gut bacteria is decreased and the abundance of enterobacteria such as enteropathogenic *Escherichia coli* is increased, implicating gut inflammation [74].

Iron is essential for efficient iodine utilization and thyroid hormone synthesis. Iron deficiency is a common finding in hypothyroidism and is diagnosed in up to 60% of these patients [75]. It does not appear to correlate with the severity of the disease and it may be caused by celiac disease, autoimmune gastritis, or other malabsorption disorders [75,76,77].

Iron deficiency may contribute to compromised thyroid hormone synthesis, storage, and secretion due to decreased oxygen transport or by impairing heme-dependent thyroid peroxidase despite adequate iodine intake. Thyroid iodine peroxidase (TPO) is located at the apical membrane of the thyrocyte and catalyzes two crucial steps in thyroid hormone synthesis: Iodination of thyroglobulin and coupling of iodotyrosine molecules. Activity of this iron-dependent enzyme can be negatively affected by iron deficiency, resulting in low thyroid hormone levels in plasma, thus increased TSH secretion and enlarged thyroid [75,78]. In 1998, Beard et al. reported significantly lower plasma T3 concentrations and a lower T4 plasma pool and T4 disposal rate in ID rats [79].

Thyroid autoimmunity, as well as ID, are prevalent among women of reproductive age and are associated with adverse pregnancy outcomes [80]. Iron deficient pregnant and non-pregnant women tend to have a markedly higher prevalence of isolated mild and severe hypothyroxinemia. Total body iron in pregnant women negatively correlates with serum TSH levels, but for non-pregnant women in this study by Yu et al., no link was found [81]. Zhang et al. investigated the association between ID and the prevalence of AITDs in 7463 pregnant and 2185 non-pregnant women of childbearing age. They concluded that the serum fT4 was lower in iron deficient women and higher in women with iron overload (IO). They found a significant higher prevalence of isolated TPO-antibody-positive women in the ID group and the prevalence is increasing with the severity of ID, suggesting that ID may be a pathogenic factor for isolated positive TPO-antibodies in women [80].

Khatiwada et al. observed significantly lower iron levels in hypothyroid Nepalese children and a strong correlation between iron deficiency and anemia with hypothyroidism. Anemic children tended to have higher TSH and iron deficient children had significantly lower fT3 levels than iron sufficient children [82].

Deficiencies of iron and iodine often coexist and high prevalence of iron deficiency among children in areas of endemic goiter has been suggested to impair the effectiveness of iodized salt programs. Several randomized clinical trials in populations at high risk for goiter and iron deficiency anemia came to the conclusion that the combined treatment with iron and iodine is superior to iodine treatment alone [78,83,84,85].

Luo et al. investigated the associations of iodine and iron with thyroid function in the US population, including 7672 participants. The serum iron level altered the association between urinary iodine concentration and thyroid function [64]. This can be explained by the crucial role of iron for TPO, which catalyzes two initial steps for the thyroid hormone synthesis, including thyroglobulin iodination and coupling reaction of iodotyrosine. In ID, TPO activity could be decreased and thus, impair iodine utilization [78]. They additionally found out that the association is sex-specific, reasoning that high urinary iodine levels increase the risk for high TSH in women, but not in men. Combined low iron and low iodine led to reduced free T3 and increased TSH. In summary, thyroid function seems to be disrupted by low levels of iron or abnormal iodine intake [64].

#### 2.3.3. Zinc

Zinc is an essential micronutrient for thyroid function and homeostasis and is required for enzyme 1,5′-deoidinase, which catalyzes the conversion of T4 to T3 and reduces metabolic rate. Superoxide dismutase enzyme contains zinc, which is considered antioxidative. Additionally, zinc is a component of thyroid hormone binding transcription factor, which is important for gene expression [86,87,88,89]. Zinc deficiency affects the thyroid gland on multiple levels: zinc deficiency impairs TRH synthesis, but also TSH, T3, and T4. Beyond that, it influences T3 binding to nuclear receptors and binding of this receptor to DNA. Possible mechanisms for zinc deficiency include impaired gastrointestinal absorption [90,91,92,93,94]. In animal studies with rats, zinc deficiency reduced free levels of T3 and T4 by around 30% [95]. In humans with zinc deficiency, TSH, T3, and T4 decrease as well and hypothyroid patients often present with reduced levels of zinc and copper. Arora et al. found a significant and positive correlation of zinc and T3, but not with TSH or T4 in their case-control study of trace elements in hypothyroidism patients [86,96]. The relationship between zinc and thyroid disorders seems to be reciprocal, considering that hypothyroidism leads to zinc deficiency and insufficient supplementation with zinc causes hypothyroidism [4,86].

#### 2.3.4. Selenium

Selenium is an essential trace mineral, involved in the immune system and in several thyroid functions. Glutathione peroxidase, deiodinase isoenzymes, and thioredoxin reductase—which protect the thyroid gland from free radicals—are just a few of the > 20 human selenoproteins. The thyroid gland contains the highest amount of selenium in the body and under conditions of deficiency, it is able to retain selenium. Although very low amounts of selenium are required for deiodinase activity, selenium deficiency can decrease synthesis of thyroid hormones and seems to have an impact on thyroid function [41,76,96,97]. The selenium content of plant based food depends on soil composition, primarily as a result of the weathering of selenium-containing rocks or volcanic activity. The availability of selenium to plants is also influenced by soil moisture. Areas including Europe, New Zealand, Sibiria, north-east, and south-central China have very low amounts of selenium in their soil. In contrast, the USA and Canada are seleniferous. In animal products, especially the inner organs contain high concentrations of selenium and the geographic variation of selenium contents in animal food is reduced by feeding supplements in commercial animal agriculture [98].

There are two main dietary forms of selenium. Selenomethionine (especially present in plant products) is taken up by intestinal methionine transporters and the absorption of selenocysteine (especially present in animal products) is poorly understood, but may be facilitated via dibasic and neutral amino acids. For selenium supplementation, inorganic selenium forms are used [99]. Selenium affects the composition and colonization of the microbiota in the intestine. Kasaikina found that selenium increases the diversity of microbiota in mice and suggested that selenium causes unique effects across microbial taxa. Gut microbiota is able to sequester selenium and limit availability for the host [100].

In thyroid disorders, selenium deficiency is a common finding, including decreased hormone and enzyme activity and reduced peripheral T3 synthesis. In patients with autoimmune thyroid diseases, selenium supplementation may reduce levels of antithyroid antibodies, improve thyroid structure, improve thyroid metabolism, and improve clinical symptoms [97,101].

#### 2.3.5. Vitamin D

The steroid hormone vitamin D is pivotal for calcium and phosphate homeostasis and is either ingested as vitamin D2 from diet or synthesized as vitamin D3 from the skin. Several studies showed that tissues that contain local 1-α-hydroxylase can produce 1α-25(OH)_2_D, inducing both autocrine and paracrine effects [76,102,103]. Vitamin D has complex effects on the immune system and is likely to affect the thyroid through its immunomodulatory effects. 1α-25(OH)_2_D is believed to protect from autoimmunity by exerting immunoregulatory and tolerogenic effects, such as impairing autoantigen presentation in dendritic-cell subsets [76,104]. Human studies concluded that hypothyroid patients often present with lower levels of vitamin D or vitamin D deficiency than healthy controls. Inverse correlations between 25(OH)D concentrations and TPOAb, TgAb titers, and TSH in hypothyroidism seem to exist as well as a positive relationship of 25(OH)D with T3 levels [76,105,106,107]. Not all studies did observe lower levels of 25(OH)D in patients with hypothyroidism [108]. Low concentrations of vitamin D in hypothyroidism could also represent a consequence of disease, rather than part of its cause [76]. However, taking into account the existing studies, as well as the low cost and minimal side effects of vitamin D, monitoring and supplementation in patients with HT may be recommended [41].

### 2.4. Probiotics

Probiotics are non-pathogenic microorganisms that can reach the colon alive, having beneficial health effects for their host [109]. In hypothyroidism and hyperthyroidism, *Lactobacillaceae* and *Bifidobacteriaceae* are often reduced. *Lactobacillus reuteri* supplementation proved to benefit the thyroid function in mice by increasing free T4, thyroid mass, and physiological parameters, like more active behavior. This effect could be triggered by interleukine-10 and subsequent enhanced T-regulatory cells [1]. In broiler chickens, two studies examined increased T3 and T4 levels after the supplementation of probiotics [110,111]. Synbiotic supplementation is a combination of pro- and pre-biotics and a recent study showed beneficial effects on patients with hypothyroidism by significantly reducing TSH, levothyroxine dose, and fatigue and increasing fT3. No influence on anti-TPO or blood pressure was observed [112]. However, when conferring animal studies, it must be considered that microbiota in different animal species are not obligatory comparable to humans. Zhou et al. found no stimulating or impairing effect of probiotics *Lactobacillaceae* and *Bifidobacteriaceae* with regards to autoimmune thyroid diseases [113].

**Figure 1:**
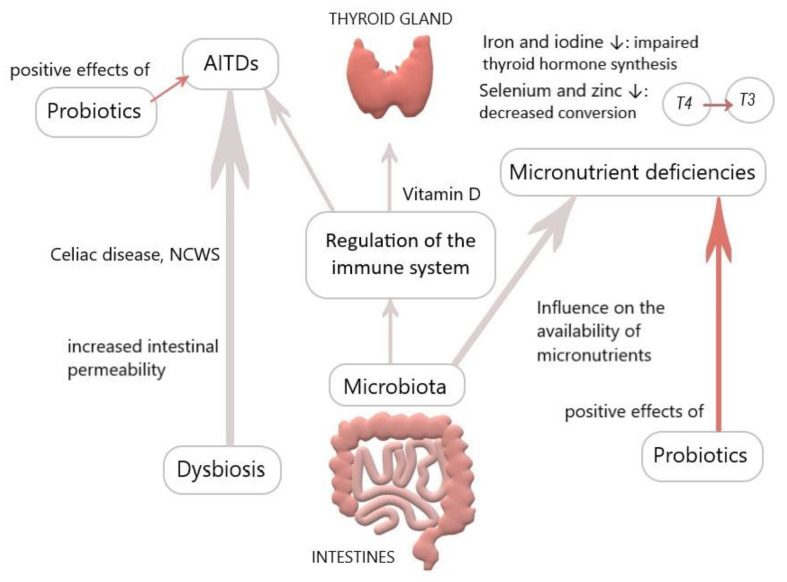
Overview of the influence of the gut on the thyroid (personal figure).

Interestingly, microbes like *E. coli* function as a reservoir for T3 by binding it to bacterial thyroid-binding hormone and are able to prevent thyroid hormone fluctuating and thus, possibly reduce the need for T4 supplementation. Spaggiari et al. investigated the influence of *Lactobacilli* and *Bifidobacteriaceae* probiotics on levothyroxine. They found a significantly lower adjustment requirement of T4 in the study compared to the control group, reasoning that microbiota modification increases levothyroxine availability and stabilizes thyroid function. They concluded that probiotics have a beneficial role in lowering serum hormone fluctuations, also considering that iodothyronines deconjugation is regulated by bacterial enzymes sulfatases and ß-glucuronidases, which could be more available due to probiotics [4,114,115]. Probiotics seem to be able to accumulate trace elements such as selenium, zinc, and copper and incorporate them into organic compounds. Considering that selenium, zinc, and probiotics operate via different pathways and all of them are favorable for the thyroid, there could be a synergistic effect for health when incorporating all of them, especially in deficient conditions [89]. Probiotics could constitute an adjuvant therapy for thyroid diseases (Figure 1); nonetheless, it must be considered that most studies on probiotics rely on animal models.

### 2.5. Bariatric Surgery

Bariatric surgery includes interventions to treat obesity-sleeve gastrectomy (SG) and Roux-en-Y gastric bypass (RYGB) are the most frequently performed interventions. Obesity implicates changes in the hypothalamic pituitary thyroid axis similarly to primary thyroid disease and body weight seems to be positively correlated with serum TSH levels. The underlying mechanisms were suggested to be influenced by adipocytokines of adipose tissue in obese patients, which have been shown to stimulate thyroid activity and thus increase TSH and T3. Despite high TSH, hypothyroidism is a common finding in obese patients, due to similar hormonal resistance as in diabetes mellitus. Additionally, changed nutritional behavior and decreased absorption of nutrients, minerals, and vitamin D after surgery through reduced gastrointestinal luminal loss may reduce thyroid hormone activity. However, results on changes in thyroid function after bariatric surgery are conflicting [116,117,118,119,120,121].

SG and RYGB significantly decrease TSH and fT3 in obese patients. Duran et al. recognized the same for SG alone, but RYGB alone increased TSH and significantly decreased fT3 and fT4 in this study. TSH increase in RYGB may be due to decreased thyroid hormones and fT3 decrease may be reactive or due to reorganization of the pituitary thyroid axis after weight loss [120].

A meta-analysis summarizing 24 studies on the issue confirmed the significant decrease of the TSH level, fT3 and T3 in obese patients after bariatric surgery, but not for fT4 and T4. The conflicting findings of former studies may be due to different surgical procedures, sample sizes, or different preoperative thyroid function, which could influence the response to bariatric surgery. For instance, in sub-group analysis of obese, euthyroid patients, a decrease in fT3 and T3 was found, but no change in TSH [119]. A further study reported a direct link between a decrease in TSH and BMI. Subclinical hypothyroidism was completely resolved in 87% of RYGB patients 1 year after surgery [122]. Other studies observed an improvement of hypothyroidism in around 35% of patients and resolution in about 15% [121]. Leptin regulates appetite and food intake and stimulates the secretion of hypothalamic hormones, such as TRH. Yu et al. demonstrated that TSH was significantly positively related with BMI, CRP, and leptin, reasoning that leptin might mediate change in thyroid function via cross talk between adipose tissue and hypothalamic pituitary thyroid axis [123]. To conclude, it is noteworthy that most studies revealed a beneficial effect of bariatric surgery on hypothyroid patients, considering thyroid function and a decrease of drug requirements.

### 2.6. Thyroid Cancer

Gut microbiota in patients with thyroid cancer and thyroid nodules presents with higher microbial richness and distinct composition compared to the healthy control group, indicating that gut microbiota is correlated with thyroid cancer and nodules. Opportunistic pathogens may colonize in patients suffering from thyroid disease [124]. Several studies have demonstrated that dysbiosis of the microbiota could be caused by inflammatory processes and diverse cancer types [125,126,127].

In thyroid cancer, relative abundance of *Clostridiaceae*, *Neisseria*, and *Streptococcus* is significantly higher, while in thyroid nodules, *Streptococcus* and *Neisseria* relatively increased in comparison to healthy control groups in one study [124]. *Clostridiaceae* apparently have carcinogenic effects [128], *Streptococcus* leads to higher risk of adenomas and carcinomas [129], and *Neisseria* has been linked to inflammatory disorders and pancreatic disease [130,131]. Considering the high prevalence in thyroid nodules and cancer, those three genera could play a role in thyroid carcinogenesis. However, *Lactobacillus* is significantly decreased in thyroid cancer and nodules groups. This genus is important for various trace elements in human cells, such as selenium, which has antioxidative and protective effects on the thyroid gland [124], implicating that lack of lactobacillus may cause higher oxidative stress in the thyroid gland. Of note, iodide is not only suggested to be an antioxidant, but also antineoplastic, anti-proliferative, and cytotoxic in human cancer. Thus, alterations of iodide expression may be associated with tumor development in a cancer-type-dependent manner [56].

Soriano et al. showed that drinking water containing at least 0.1% molecular iodine or iodide has antineoplastic effects and increases the expression of peroxisome proliferator-activated receptor type γ (PPAR γ), triggering apoptosis pathways in mammary pre-cancer and cancerous cells, but not in normal mammary gland [132].

## 3. Conclusions

There is accumulating data that a strong thyroid–gut axis exists. It appears to display a not well known but important correlation regarding the effect of the gut bacteria on the immune system and thyroid function. Furthermore, there is higher prevalence of the coexistence of thyroid and gut related diseases, just as Hashimoto’s Thyroiditis/Graves’ Disease and Celiac Disease/Non-celiac wheat sensitivity. Dysbiosis is a common finding in thyroid disorders. On the one hand, it alters the immune response by promoting inflammation and reducing immune tolerance, damaging the intestinal membrane and causing an increase in intestinal permeability, which again leads not only to a high exposure of antigens, but also local inflammation. On the other hand, it can directly impact thyroid hormone levels through its own deiodinase activity and the inhibition of TSH. Gut microbiota also influences the absorption of minerals that are important to the thyroid, including iodine, selenium, zinc, and iron. All of them are essential for thyroid function and there is a clear link between thyroid dysfunction and altered levels of these minerals. For example, iodine deficiency may lead to goiter, presumably thyroid nodules, and even follicular thyroid cancer. High iodine intake can either induce hypothyroidism or hyperthyroidism in susceptible patients. Iron is essential for bacterial growth, iron availability influences the composition of the microbiota, and at the same time, the microbiota influences iron availability. Iron is vital for efficient iodine utilization and thyroid hormone synthesis and ID could cause thyroid disorders, including impaired thyroid hormone synthesis, storage, and secretion.

Probiotics have shown beneficial effects in thyroid diseases and are able to have a positive effect on trace elements such as selenium, zinc, and copper. Additionally, microbes function as a reservoir for T3 and are able to prevent thyroid hormone fluctuating and thus may be able to reduce the need for T4 supplementation. Probiotics could constitute an adjuvant therapy for thyroid diseases. However, most studies on probiotics rely on animal models, therefore well-designed human studies are needed to further elucidate the importance of the thyroid–gut axis and the possibilities of intervention.

Taking into account the various potential effects of the microbiota and micronutrients on thyroid functions and medications, novel therapeutic strategies for the management of thyroid diseases could be established and more specifically adjusted to patients, depending on their gut bacteria composition. Future adequately powered human studies would be necessary to evaluate the impact of gut microbiota on thyroid function and diseases.
